# Variability and Belief in Karma: Perceived Life Variability Polarizes Perceptions of Behavior–Outcome Valence Consistency

**DOI:** 10.3390/bs15040400

**Published:** 2025-03-21

**Authors:** Liying Jiao, Zhen Guo, Jinzhe Zhao, Yan Xu

**Affiliations:** 1 Department of Psychology, School of Humanities and Social Sciences, Beijing Forestry University, Beijing 100083, China; 2 School of Psychology, Nanjing Normal University, Nanjing 210097, China; zhenguo@njnu.edu.cn; 3 Beijing Key Laboratory of Applied Experimental Psychology, National Demonstration Center for Experimental Psychology Education (Beijing Normal University), Faculty of Psychology, Beijing Normal University, Beijing 100875, China; zhaojz@mail.bnu.edu.cn

**Keywords:** karma, moral behavior, future experiences, perceived life variability

## Abstract

This article explores people’s karma belief on the subjective probabilities of future chance events and how perceived life variability influences the expectations of behavior–outcome relationships through three studies. Study 1 used two experiments (Study 1a and 1b) and found that individuals believed that there is a causal association between moral actions and future experiences. People tended to make karmic forecasts that predicted a luckier future (reflected in probability judgments of lucky and unfortunate chance events) for a person who behaved morally than for one who behaved immorally. Finally, we found that individuals rely more heavily on belief in karma (i.e., stronger beliefs about the valence consistency of behaviors and outcomes) when they perceive greater life variability in their lives (Study 2), suggesting that the perceived life variability is a factor in using karma to make predictions.

## 1. Introduction

People are often exposed to events for which the outcomes are commonly attributed to good or bad fortune (Teigen et al., 1999). In many people’s minds, the probability of a lucky outcome is not random; it can be greatly affected by other factors (Kulow & Kramer, 2016; Pepitone & Saffiotti, 1997). According to the good-behavior–good-outcome association (Converse et al., 2012), researchers suggest that people may believe that their fortune will be influenced by moral behavior. In other words, past moral behavior increases the likelihood of experiencing valence-congruent outcomes. For instance, if a person encounters a sudden illness, his or her friends may be more likely to believe that he/she will be able to recover if he/she has been very kind to others in the past. This causal belief in the connections between actions and future experiences in supernatural justice is named “karma” or “belief in karma”, which means “good actions cause good things to happen and bad actions cause bad things to happen, even when no physical connection is discernible between actions and experiences” (White & Norenzayan, 2019, p. 2). While substantial evidence suggests that belief in karma exists in religion, knowledge about the psychology of karma and its causes is still limited (White et al., 2019b). The key questions that we focused on are (1) whether individuals engage in making karmic forecasts and (2) how this process can be affected by individuals’ perceived life variability.

The concept of karma has a deep impact on people’s psychology. Researchers have found that people tend to make karma attributions even though they have no explicit religious beliefs or they deny that they believe in karma (Banerjee & Bloom, 2017; White et al., 2019b). Belief in karma effectively connects individuals’ longevity, wealth, pro-social treatment by others, suffering, misfortune, failure, and accidents with their previous good and bad deeds. Since people want to obtain positive outcomes for themselves, belief in karma has the function of encouraging people to act morally and of dissuading people from performing evil acts (S. J. Tang & Guo, 2019; White et al., 2019a).

Moreover, according to the compensatory mechanism of belief in karma, belief in karma helps to maintaining people’s psychological well-being when their need to feel in control of their lives is threatened (White & Norenzayan, 2019). For example, research has found that belief in karma promotes Indian consumers’ life satisfaction (Roy et al., 2020), and individuals who scored high on the Karma-Yoga scale performed well on emotional intelligence tests (Mulla & Krishnan, 2007). Belief in karma fosters people’s optimistic attitude about the future, provides people with a sense of an orderly and balanced world, and allows them to rationalize the suffering in life (Dhiman, 2018; Kisala, 1994; White & Norenzayan, 2019). Therefore, exploring belief in karma and its influencing factors plays an important role in understanding people’s mental health.

Since belief in karma involves a causal relationship between moral behavior and expectations (Daniel, 1983; Wadley, 1983; White et al., 2017; White & Norenzayan, 2019, 2022), it provides a psychological framework for interpreting and predicting events. People use karma to interpret why some people have good (e.g., wealth, status, and pro-social treatment from other people) or bad (e.g., illness, accident, failures) fortune (Liamputtong & Suwankhong, 2016; P.-L. Tang et al., 2016). Previous research on karma primarily focused on retrospective evaluations; however, researchers have also emphasized that exploring whether the belief in karma also extends to predicting future outcomes is also important (Banerjee & Bloom, 2017; Mata & Simão, 2020; White & Norenzayan, 2019). Such a concern for karma may establish a real-time expectation of outcomes (Callan et al., 2013) and help expose people’s underlying motivations when facing uncertainty, which may also influence individuals’ future actions and decisions (Converse et al., 2012). For instance, people tend to predict a luckier future for individuals who purchase a product associated with a highly moral person, in comparison to those who unknowingly purchase a product associated with a highly immoral person (Stavrova & Meckel, 2017). Therefore, we tested the belief in karma in forecasting. We proposed that people make karmic forecasts about how likely others are to have positive or negative experiences.

Additionally, much of the existing research on supernatural moralizing systems, such as belief in karma, has been conducted within a Western, Educated, Industrialized, Rich, and Democratic (WEIRD) background, which may limit the comprehensiveness of the investigation into belief in karma (Willard et al., 2020). Enriching the sample types may help us to comprehend how widely the belief is held and understand it deeply. Therefore, this study was conducted in Chinese contexts, which are generally regarded as non-Western culture (e.g., McHugh et al., 2023; Muthukrishna et al., 2020). While some studies have examined belief in karma in some non-Western populations (e.g., Willard et al., 2020; White et al., 2021), studying karma beliefs and their influencing factors in Chinese samples remains important due to China’s unique culture, influenced by Confucianism and Buddhism. These belief systems emphasize an indigenous understanding that aligns with the belief in karma (Leung, 2012).

### 1.1. Perceived Life Variability and Belief in Karma

Variability refers to changes or fluctuations occurring over time. People experience variability in their daily lives (e.g., changes in weather or commodity prices) and are exposed to this variability in objective representation (e.g., weather reports and economic news). Many factors can stimulate individuals’ perceived variability in their lives, including the global environment, the mobility of enterprise personnel, the frequent changes in their work, the change in housing prices, the fluctuation of interpersonal relationships, their lifestyle, and the living environment. The factors that may cause individuals’ perceived life variability can even result in personality and mental health changes (Haehner et al., 2022; Luhmann et al., 2012; Shiner et al., 2017). Moreover, researchers have found that, in some cases, subjective perception has a greater impact on individuals’ psychological state and behavior than objective reality (e.g., Broadway & Haisken-DeNew, 2019; De Witte et al., 2010; Lewin, 1939). Therefore, it is important to study how perceived life variability affects individuals’ psychology.

Variability is different from lack of control and uncertainty, which refer to individuals’ psychological state of being unable to predict or influence future outcomes; life variability focuses on the broader perception of the inherent unpredictability and fluctuation of life events. High perceived life variability could induce people’s sense of unpredictability, lack of order, uncertainty, threat, and a reduced sense of control (Ding & Savani, 2020). Compensatory control theory (CCT) suggests that people prefer a world that is predictable, orderly, controllable, stable, and meaningful (Kay et al., 2010, 2009; Laurin et al., 2013; Laurin & Kay, 2017; Rothbaum et al., 1982; Vail et al., 2010). Consequently, the motivation to control and predict the world prompts people to use compensatory strategies in response to events that reduce personal control (Landau et al., 2015). Lines of research have shown that supernatural justice beliefs have a compensatory function in people’s lives (Fritsche et al., 2008; Kay et al., 2008; Liu & Liao, 2020). The belief in unseen supernatural forces such as karma provides a way to satisfy the need to see the world as just, which can reduce uncertainty and provide people with a sense of control (White & Norenzayan, 2019). People are more willing to rely on karma when they anticipate an uncertain outcome that is beyond their control (Converse et al., 2012).

Moreover, people who sense greater perceived life variability also consider their society to be more vulnerable, meaning that it faces more threats that are difficult to handle (Ding & Savani, 2020). Greater perceived life variability leads people to feel greater threat (Brown et al., 2007; Ding & Savani, 2020; Thornton et al., 2014) and a greater desire for control (DC, Ding & Savani, 2020), reflecting people’s need to control their environment (Burger & Cooper, 1979; Keinan, 2002). Evidence from extensive research suggests that individuals may resort to belief in karma and behavior in conditions of threat. For example, mortality salience leads Asian students to believe in karma (C. L. Yen, 2012). The desire for control can also increase the belief in magical thinking (e.g., karma belief), leading people to see a causal pattern in a series of causally unrelated events (Friedland & Keinan, 1991; LeBoeuf & Norton, 2012; Stavrova & Meckel, 2017) and relieving the anxiety caused by perceived life variability. In addition, the theory of locus of control can underpin the notion that perceived life variability enhances belief in karma. Locus of control reflects individuals’ beliefs in the extent to which they have control over events in their lives (Galvin et al., 2018). People with an internal locus of control believe that what happens to them can be determined by themselves, while individuals with an external locus of control tend to attribute their life outcomes to external forces like chance factors or powerful others (Guo et al., 2021). Furthermore, environments that stifle individuals’ control have the potential to influence their locus of control (Johnson et al., 2016). Hence, the unpredictability and uncertainty that people experience due to high perceived life variability might bring them a feeling of not being in control of events that happen to them, thereby increasing their external locus of control, which is also related to supernatural beliefs (Khanna & Khanna, 2007; Randall & Desrosiers, 1980). Therefore, we propose that the perceived life variability can further people’s belief in karma.

### 1.2. Current Research

Based on previous research, the goal of this study is to examine whether karmic forecasting exists within Chinese culture, specifically whether individuals expect the likelihood of a person experiencing future positive (or negative) events to be influenced by that person’s previous moral/immoral behavior and how perceived life variability affects this process. The current study includes three studies. We asked participants to estimate the likelihood of positive (e.g., have a good luck) and negative (e.g., have a bad luck) chance events occurring to people who have previously behaved morally, in comparison to those who have exhibited immoral behavior (Study 1a, 1b). We also discussed the role of perceived life variability in promoting the belief in karma (Study 2). All studies were approved by the institutional ethics committee of the authors’ affiliated institution.

## 2. Study 1a

The goal of this study was to extend previous research (e.g., Converse et al., 2012; Mata & Simão, 2020) by examining whether individuals in the Chinese context hold the belief in karma to predict future events. We expected that the perceived likelihood of a good/bad outcome would be affected by the moral worth of the target person’s behavior, such that participants would consider that a person who performed a good deed has a higher chance of enjoying a fortunate outcome than does a person who performed a bad deed.

### 2.1. Method

#### 2.1.1. Participants

For this study, we recruited 148 participants from a Chinese psychology class. Of these, 13 failed either the manipulation check or data-quality check questions (which required them to recall important details in the material they were provided), and 4 were aware of the actual purpose of the experiment; consequently, they were excluded from the analyses, resulting in a final sample of 131 participants (79 females, 48 males, 4 did not report their gender) with a mean age of 20.20 years (*SD* = 2.10). Eight of the selected participants did not report their age for the experiment. Participants were volunteers in Study 1a. Participants of all studies of the present research were required to fill out the informed consent form and could freely withdraw at any time during the experiment.

#### 2.1.2. Materials and Procedure

Participants were randomly assigned to one of two experimental conditions (protagonist’s behavior: moral vs. immoral) using a between-subject design.

The participants were informed that the study concerned the accuracy of individuals’ memories of current events, and they were asked to comment on a news article copied from a newspaper. Adobe Photoshop CS6 software was used to process the materials so that they resembled a genuine newspaper article. The news story concerned Mr. Zhou, a kindergarten teacher and administrator, who was severely burned while rescuing four children from a fire (or, in the alternate scenario, seriously abused four children, scalding one so badly with boiling water that the child became comatose). The materials in the two conditions were as consistent as possible in terms of form, number of words, and perceived truthfulness, in order to reduce the effect of confounding variables. Afterward, to ensure that they had read the news carefully, participants were asked to recall the name of the teacher and what he had done.

Then, participants were asked to indicate the probability that Mr. Zhou would experience each of several events at some point in his life, ranking the likelihood of each event occurring using a scale ranging from 1 (*far below average*) to 9 (*far above average*). These events were modified from items used by Stavrova and Meckel (2017). There were nine positive events (e.g., *be very fortunate in life*) and nine negative events (e.g., *be victim of a natural disaster*) in Study 1a (Cronbach’s α = 0.89). The order in which the events were listed varied randomly among participants. The probability judgments for the positive and negative events (reverse scored) were averaged into scales of good luck events.

Participants were asked to answer the question “*To what extent do you think the report is true*” using a scale ranging from 1 (*completely untrue*) to 5 (*completely true*) and to rate the morality of Mr. Zhou’s behavior from 1 (*very immoral*) to 5 (*very moral*). Finally, the participants were asked whether they knew the purpose of this experiment and, if so, to write it down.

### 2.2. Results and Discussion

#### 2.2.1. Manipulation Checks

Results showed that Mr. Zhou’s behavior was perceived as more moral in the rescuing children condition (*n* = 70, *M* = 4.43, *SD* = 0.53) than in the abusing children condition (*n* = 61, *M* = 1.41, *SD* = 0.62; *t* (129) = 30.24, *p* < 0.001, 95% CI [2.82, 3.22], Cohen’s *d* = 5.24), and there was no significant difference between the rescuing children condition (*n* = 70, *M* = 4.00, *SD* = 0.61) and the abusing children condition (*n* = 61, *M* = 3.89, *SD* = 0.76) in the perceived truthfulness of the news, *t* (129) = 0.96, *p* = 0.34, 95% CI [−0.12, 0.35], Cohen’s *d* = 0.16, showing that the experimental manipulation was successful.

#### 2.2.2. Subjective Probability Judgment for Good Luck Events

**Figure 1:**
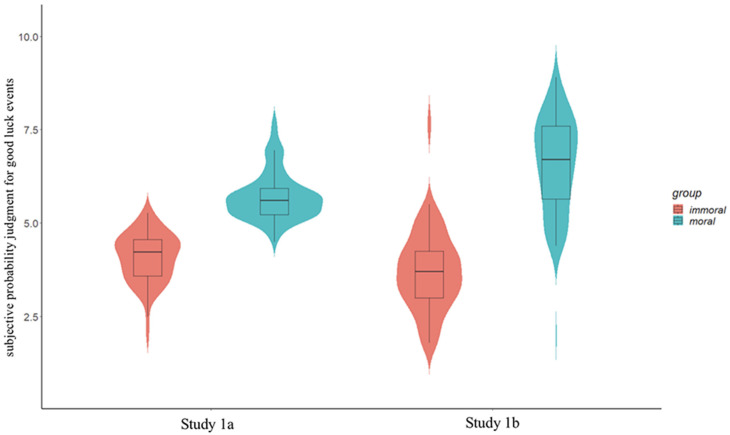
The effect of the source of a person’s morality on subjective probability judgments for good luck events, Studies 1a and 1b.

Figure 1 and Table 1 elucidate the effect of the source person’s morality on the probability judgments for good luck events in Study 1a. The results show that participants believed a person who rescued children from a fire was more likely to experience good luck events (*M* = 5.71, *SD* = 0.64) than someone who abused children (*M* = 4.06, *SD* = 0.66), *t* (129) = 14.49, *p* < 0.001, 95% CI [1.42, 1.87], Cohen’s *d* = 2.54. We performed a sensitivity analysis using G*Power 3.1 (Faul et al., 2007) to estimate the effect size that the study was able to detect with *N* = 131, a power of 1 − β = 0.85, and α = 0.05 (two tails). The findings suggest that the minimum effect size for which the independent *t*-tests were sufficiently sensitive was *d* = 0.53. The effect size in Study 1a was larger than the minimum effect size.

Thus, Study 1a provided initial support for the hypothesis that people hold the belief in karma that good people are more likely to experience positive outcomes and bad people are more likely to encounter negative outcomes. However, the item “be victim of a natural disaster” showed no significant difference between two conditions. This can be attributed to the scope of the impact on the victims. Specifically, natural disasters typically affect large groups of people simultaneously. In contrast, other events typically target individuals. The manipulated conditions in this study primarily focused on an individual’s moral/immoral behavior. Therefore, events that are more likely to happen to an individual are more easily perceived as being influenced by that person’s moral or immoral behavior.

## 3. Study 1b

The goal of Study 1b was to test whether the same effect arises when the person conducted other moral (vs. immoral) behaviors.

### 3.1. Method

#### 3.1.1. Participants

Previous research (e.g., Leung, 2012; S. J. Tang & Guo, 2019) suggests that belief in karma or similar concepts of retribution is a common aspect of Chinese culture. Additionally, our preliminary survey (*N* = 290) found a mean score of 3.53 for belief in karma, indicating a moderate level of belief that karma exists. Therefore, we estimated a medium-sized effect (*d* = 0.50) for a *t*-test. Using this estimate, a priori power analysis indicated that we should target 146 participants to obtain 85% power at a 0.05 alpha level (G*Power V3.1; Faul et al., 2007). Relying on the same power analysis, in this study, 170 participants were recruited (100 females, mean age 30.34 years, *SD* = 8.79) through *Sojump*, a Chinese online platform where individuals can complete surveys and participate in psychological experiments.

#### 3.1.2. Materials and Procedure

The participants were informed that the study concerned individuals’ views of a news event. Participants read a story about what Mr. Zhou had done in early February 2020, when the whole country was entering a state of emergency in response to the COVID-19 outbreak. In the moral example, Zhou took the lead to form a team of six people and traveled for 36 h to deliver 100 tons of fresh vegetables to Wuhan, ensuring food supply for numerous families during the outbreak. In the immoral example, Zhou was suspected of selling more than 10,000 fake and inferior masks in many places, and some of them even arrived at local hospitals, leading to dozens of medical staff being quarantined. The materials in the two conditions were as consistent as possible in terms of form, number of words, and the social impact that protagonists made, in order to reduce the effect of confounding variables.

Then, participants were asked to indicate the probability that Mr. Zhou would experience each of several events at some point in his life, ranking the likelihood of each event occurring using a scale ranging from 1 (far below average) to 9 (far above average). There were five positive events (i.e., *get a better job*; *have good luck; receive CNY 1000 as a gift from a strange*; *be very fortunate in life*; *always “be in the right place at the right time”*) and five negative events (i.e., *fall victim to a brutal assault*; *contract the virus*; *be bullied by colleagues*; *find mobile phone was stolen*; *have bad luck*) in Study 1b (Cronbach’s α = 0.91). The order in which the events were listed varied randomly among participants. We reduced the number of event items to simplify the task for participants and reduce their burden. Events that were most clearly representative of common life experiences and highly relevant to our research question were retained, with some appropriate adjustments made. The probability judgments for the positive and negative events (reverse-scored) were averaged into scales of good luck events.

Finally, participants rated the influence of the protagonist’s behavior on society from 0 (*not at all*) to 10 (*extremely*), and the moral level of the protagonist’s behavior from 1 (*very immoral*) to 7 (*very moral*). Participants were paid CNY 1.5 after completing all tasks.

### 3.2. Results and Discussion

#### 3.2.1. Manipulation Checks

The manipulation check indicated that the protagonist was perceived as more moral in the donated materials example (*n* = 79, *M* = 6.24, *SD* = 0.95) than in the sold fake masks example (*n* = 91, *M* = 1.84, *SD* = 1.70; *t* (145.04) = 21.25, *p* < 0.001, 95% CI [4.00, 4.82], Cohen’s *d* = 3.20). There was no significant difference between the moral example (*M* = 7.82, *SD* = 1.70) and the immoral example (*M* = 8.25, *SD* = 1.88) in the influence of the protagonist’s behavior on society, *t* (168) = −1.56, *p* = 0.12, 95% CI [−0.98, 0.12], Cohen’s *d* = −0.24, showing that the experimental manipulation was successful.

#### 3.2.2. Subjective Probability Judgment for Good Luck Events

Participants believed a person who engaged in moral behavior (donated materials) was more likely to experience good luck events (*M* = 6.55, *SD* = 1.30) than someone who behaved immorally (sold fake masks; *M* = 3.73, *SD* = 1.09), *t* (168) = 15.37, *p* < 0.001, 95% CI [2.46, 3.18], Cohen’s *d* = 2.36; see Figure 1.

Thus, Study 1b provided further support for the existence of the belief in karma, which indicates that people believe there is a causal association between luck and morality. The results in Study 1 were consistent with previous findings on belief in deservingness that positive (negative) outcomes happen to good (bad) people (Feather, 1992; Lupfer & Gingrich, 1999) and provided further evidence indicating that event–outcome valance consistency is not only for judgments of what has already happened but also for expectations of future events. In Study 2, we explored the factors influencing belief in karma. Specifically, we examined whether perceived life variability could enhance individuals’ belief in karma.

## 4. Study 2

Study 2 aimed to replicate our previous studies on the belief in karma and explore whether perceived life variability moderates the extent of belief in karma. We hypothesized that perceived life variability could polarize people’s perception of past moral behavior, thereby increasing the likelihood of experiencing valence-congruent outcomes.

### 4.1. Method

#### 4.1.1. Participants

We estimated a medium-sized effect (*f* = 0.25) because we were uncertain what effect sizes we would observe. Using this estimate, a priori power analysis indicated that we should target 146 participants to obtain 85% power (G*Power V3.1; Faul et al., 2007). One hundred and ninety-eight Chinese participants were recruited through Credamo (www.credamo.com), an online questionnaire survey platform widely used in academic research. Since all data were based on self-report, to control the Common Method Variance (CMV, Podsakoff et al., 2003; Tehseen et al., 2017), participants were asked to complete the study in two time periods with a one-week interval. Participants whose answer time was too short (the answer time in the first period was less than 100 s, or it was less than 60 s in the second period) were excluded. The final sample comprised 149 people (41 female, mean age 24.24 years, *SD* = 7.12).

#### 4.1.2. Materials and Procedure

Participants completed the following measures in period 1.

Perceived Life variability. Participants completed a three-item measure of perceived life variability in their lives (e.g., “How variable are most things in your life?”; α = 0.79; adapted from Ding & Savani, 2020). This was rated on a 7-point scale (1 = *not at all*, 7 = *extremely*).

Control variables. Participants provided demographic information, including age, gender, educational background, and religious background. We also controlled individuals’ justice beliefs. Participants completed a seven-item measure of Belief in a Just World using a 6-point scale (1 = *strongly disagree*, 6 = *strongly agree*), for example, “I feel that people get what they deserve” (α = 0.83; adapted from Lipkus et al., 1996).

Participants completed a fortune prediction task that was identical to that of Study 1b in period 2. There were five positive and five negative events, α = 0.95. In addition, participants had to rate the perceived truthfulness of the news from 1 (*completely untrue*) to 5 (*completely true*) and the moral level of the protagonist’s behavior from 1 (*very immoral*) to 7 (*very moral*); both served as manipulation checks. At each period, participants would receive CNY 2 as a reward after completing the survey.

### 4.2. Results and Discussion

The protagonist was perceived as more moral in the moral example (*M* = 6.03, *SD* = 1.45) than in the immoral example (*M* = 2.27, *SD* = 1.90; *F* (1,147) = 182.23, *p* < 0.001, η^2^ = 0.55). There was no significant difference between the moral example and the immoral example in the perceived truthfulness of the news (*M_moral_* = 4.29, *SD_moral_* = 0.83; *M_immoral_* = 4.16, *SD_immoral_* = 0.84; *F* (1,147) = 0.98, *p* = 0.32, η^2^ = 0.007), indicating that the experimental manipulation of the protagonist’s behavior (moral vs. immoral) was successful.

The results showed that participants believed a person who engaged in moral behavior was more likely to experience good luck events (*n* = 72, *M* = 7.11, *SD* = 1.36) than someone who behaved immorally (*n* = 77, *M* = 3.62, *SD* = 0.94), *t* (125.40) = 18.15, *p* < 0.001, 95% CI [3.11, 3.87], Cohen’s *d* = 2.99.

**Figure 2:**
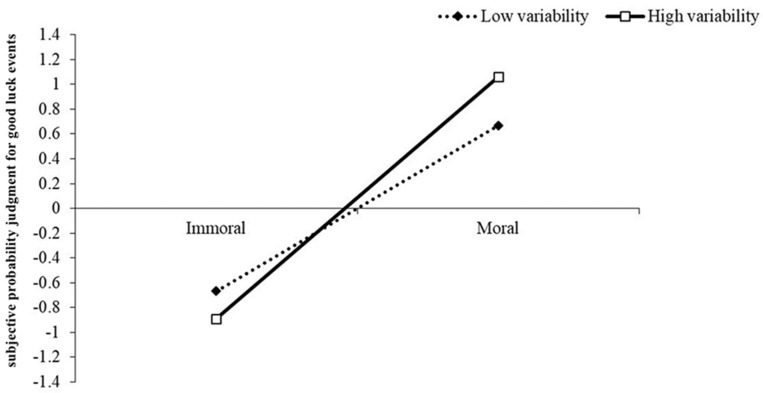
The moderating effect of perceived life variability in the relationship between moral actions and future luck experiences, Study 2.

We next tested whether there is a moderating effect of perceived life variability on the causal connection between moral actions and future experiences using the SPSS 25 PROCESS macro (Hayes, 2013, Model 1) with 5000 bootstrapped samples. We set the group condition as the independent variable (1 = moral, 0 = immoral); perceived life variability as the moderator variable; gender, age, religion, education, and belief in a just world as covariates; and good luck outcomes as the dependent variable. The results showed that different groups significantly predicted the subjective probability judgment (β = 1.66, *t* = 18.78, *p* < 0.001, 95% CI [1.49, 1.84]). Perceived life variability was not significant in predicting the probability judgment (β = −0.10, *t* = −1.51, *p* = 0.13, 95% CI [−0.24, 0.03]). The interaction between perceived life variability and the group condition was significant (β = 0.29, *t* = 3.24, *p* < 0.01, 95% CI [0.11, 0.46]). Simple effect analysis showed that the valence consistency of moral actions and future experiences was stronger in situations of high perceived life variability (+1 *SD*, β = 1.95, *SE* = 0.13, *t* = 15.60, *p* < 0.001, 95% CI [1.71, 2.20]) than in those of low perceived life variability (−1 *SD*, β = 1.33, *t* = 9.85, *SE* = 0.14, *p* < 0.001, 95% CI [1.07, 1.60]); see Figure 2.

Study 2 showed, once again, that people who believe in karma predict that the person who donated materials would be more likely to experience more good luck events in the future than the person who sold fake masks. Furthermore, perceived life variability amplified this difference. The greater unpredictability of life that participants perceived, the stronger they believed that moral actions influence future experiences.

## 5. General Discussion

We conducted three studies that addressed whether participants connect morality with good luck and whether perceived life variability increases the belief in karma. Participants predicted a luckier future (as measured by comparing the perceived likelihood of experiencing positive or negative events) for people who engaged in moral behavior (rescued children in Study 1a, donated materials in Study 1b and Study 2) than those who behaved immorally (abused children in Study 1a, sold fake masks in Study 1b and Study 2). Study 2 provided evidence that the perceived life variability had a moderating effect between moral behavior and outcome judgment, indicating that greater perceived life variability facilitates people’s belief in karma.

These results added to the current research on lay karma beliefs via psychological experiments. Although the general concept of karma exists in most cultures (Banerjee & Bloom, 2017; Davidson et al., 2005), it seems that many people do not fully understand the role of belief in karma in their interactions with others and in daily life (Allen et al., 2015). This study replicated the role of belief in karma in forecasting future experiences in Chinese culture even in the absence of explicit religious beliefs. Before Buddhism influenced Chinese philosophy in the first century C.E., the concept of *baoying* (*报应*), which was similar to but more complex than the notion of belief in karma, had existed in Chinese culture (A. S. Levy, 1995). The core of the concept of *baoying* is the concept of *bao*, which refers to a response or retribution in social relations. The norm of reciprocity (*bao*) is a powerful and perpetual principle of maintaining social relations (*guanxi*, *关系*) in China (Hwang, 1987, 2012), leading people to believe that what they do will be reciprocated in a similar manner. This explains the Chinese cultural foundation of the concept of karma. Previous studies also found that using karma for interpreting future events is also prevalent in Asian cultures (e.g., B. R. Levy et al., 2009; C. Yen & Cheng, 2010). On this basis, our studies demonstrated that people’s belief in karma can also shape their anticipation of future experiences (either positive or negative) based on their moral behavior in Asian culture.

In addition, one novel finding of this study is that perceived life variability appears to shape individuals’ adherence to belief in karma, which suggests that one’s good or bad behavior can cause his/her good or bad outcomes. This, in turn, may lead individuals to engage in karmic investments that involve acting more pro-socially when facing important life outcomes that are uncontrolled (Converse et al., 2012). People’s lives are often marked by uncertainty and instability. For example, during the COVID-19 outbreak, when Study 2 was conducted, factors such as fluctuating prices, city lockdowns, economic instability, and other social phenomena contributed to individuals perceiving high variability in their lives. In fact, an increasing number of social psychologists began to focus on individual psychology and social mentality amidst such uncertainty, using social psychological theorizing and methods to explore how people adjust and understand the precarity of the world (e.g., Adam-Troian et al., 2023; Coultas et al., 2022; Griskevicius et al., 2013). Therefore, the current study showed that perceived life variability may provide a new perspective on the social psychology of how people’s belief in karma performs its compensation function, which could contribute to addressing the challenges that arise in a rapidly changing and unpredictable world, ultimately promoting the well-being of both individuals and society during uncertain times.

In interpreting the results of this study, it is crucial to consider how morality is understood differently across cultural contexts. Our research focused on serious harmful actions (e.g., assault, fraud), which are typically regarded as “immoral” behaviors in Western culture. However, in Chinese culture, the term “不道德” (*bu daode*) does not always solely refer to harm. It can sometimes overlap with behaviors that are typically seen as “uncivilized” (不文明, *bu wenming*), such as cutting in line or being excessively loud in public spaces (Buchtel et al., 2015; Dranseika et al., 2018). This highlights an important cultural phenomenon—the Chinese understanding of “不道德” (*bu daode*) can be broader, encompassing behaviors that may not be harmful but are socially disruptive. Consequently, belief in karma in China may extend beyond traditional moral violations to include these more *bu wenming* behaviors. Understanding this cultural variability provides a unique lens through which we can examine the relationship between belief in karma and perceptions of morality.

### Limitations and Future Directions

Variability is an important aspect of socioecological psychology (Ding & Savani, 2020; Oishi, 2014). It can arise not only from environmental factors like climate change but also from personal life fluctuations, such as residential mobility. In this study, we only focused on individuals’ perceived life variability. Future empirical research could explore external environment variability, utilizing preregistered manipulation. In addition, there are still some limitations in the design of the experimental materials. The study only tested the perceived truthfulness of the news or the social impact that protagonists made in different conditions; future studies should consider more aspects to increase the internal validity.

This study is a preliminary exploration of how perceived life variability influences belief in karma. To further contribute to the field, we encourage further studies to delve deeper into various types of life variability, such as economic uncertainty, interpersonal relationship changes, health fluctuations, and other relevant factors, to examine their potential effects on belief in karma. Moreover, this study did not explore the psychological mechanisms of why perceived life variability polarized belief in karma. Possible explanations include perceived threats and a desire for control, which aligns with the compensatory control theory. This theory suggests that when individuals feel a lack of control, they tend to resort to magical thinking, believing in cause-and-effect relationships between seemingly random behaviors (Kay et al., 2009; Landau et al., 2015). Therefore, the effect of perceived life variability on compensatory behaviors and motivational beliefs (e.g., belief in karma) may involve a stable pathway driven by perceived threats and a desire for control. We also advocate for future research to explore potential mediating or moderating mechanisms, which might affect the strength or direction of the relationship between perceived life variability and belief in karma.

Moreover, another limitation of the current study is that some of the events included in the list of good and bad luck may be perceived as rationally connected to individuals’ moral actions, rather than being purely based on luck. For instance, events such as *receiving CNY 1000 as a gift* for moral actions or *being bullied by colleagues* for immoral behaviors can be seen as reflecting societal values rather than representing luck-based outcomes. To address this, we conducted additional analyses that only included events more directly linked to luck. The results showed that in Study 1a, participants believed a person who engaged in moral behavior was more likely to experience good luck events (*M* = 5.55, *SD* = 0.70) than someone who behaved immorally (*M* = 4.28, *SD* = 0.68), *t* (129) = 10.49, *p* < 0.001, 95% CI [1.03, 1.51], Cohen’s *d* = 1.84. Similarly, in Study 1b, participants rated moral behavior as more likely to result in good luck (*M* = 6.50, *SD* = 1.32) compared to immoral behavior (*M* = 3.84, *SD* = 1.21), *t* (168) = 13.67, *p* < 0.001, 95% CI [2.28, 3.05], Cohen’s *d* = 2.10. These findings suggest that moral behavior is associated with perceptions of good luck, but future research could benefit from exploring events that more closely align with the traditional concept of luck or karma. Furthermore, the perceived degree of morality/immorality of the manipulated condition stories may not have been fully balanced. Specifically, participants seemed to view the negative events as more extreme than the positive ones; this imbalance may influence participants’ judgments. In future studies, it would be beneficial to pre-test the perceived degree of morality/immorality.

In addition, the results are of limited generalizability. Specifically, the participants were recruited only from China; it is also imperative for research to consider the cultural variations while delving into topics concerning peoples’ beliefs or cognitions (Willard et al., 2020). On one hand, individuals’ belief in karma could be shaped by culture (White et al., 2017; White & Norenzayan, 2019), and moral values may vary across cultures (Graham et al., 2016); hence, anticipations regarding the outcomes of future events concerning individuals’ moral actions may exhibit cultural specificity. Furthermore, as previously elucidated, the moderating effect of perceived life variability on individuals’ belief in karma could be attributed to personal control, which could be influenced by cultural characteristics. For example, evidence has supported that there is a causal bidirectional relationship between cultural tightness–looseness, which refers to “the strength of social norms and degree of sanctioning within societies” (Gelfand et al., 2006, p. 1226), and individuals’ personal control (Ma et al., 2022). In addition, belief in karma also correlates with individuals’ political attitudes (e.g., Östervall, 2022), which also need to be considered in studies of belief in karma. Therefore, we encourage future studies to diversify sample sources for enhancing the generalizability of the findings.

It is important to note that although belief in karma has a positive function of inspiring people to act morally (e.g., more generous in the repeated dictator game, White et al., 2019a), belief in karma might also have a darker side; for example, belief in karma predicted worse health after a tsunami disaster (Banerjee & Bloom, 2017; B. R. Levy et al., 2009). For some individuals, belief in karma may provide a pessimistic thinking style that bad outcomes in life cannot be avoided. The present research provided evidence that people’s cognitive bias toward anticipating the probability of good or bad fortunes is not random; it can be greatly affected by previous moral behavior. Future studies can not only explore the situational factors that influence the belief in karma but also discuss the boundary conditions of good and bad effects of belief in karma.

## 6. Conclusions

The current study presents evidence supporting the existence of belief in karma; specifically, people tended to predict a more fortunate future for individuals who engaged in moral behavior than immoral behavior. The results also shed light on the moderating role of perceived life variability in the behavior–outcome valence consistency effect, suggesting that individuals who perceive greater life variability are more likely to expect that moral behaviors strongly influence future experiences. Our findings offer additional insights into the intricate interplay between individuals’ life perceptions and their belief in karma concerning the impact of moral behavior on future outcomes.

## Figures and Tables

**Table 1:** Subjective probability judgments for good luck events in different conditions, Study 1a.

Events	Moral		Immoral				
	*M*	*SD*	*M*	*SD*	*t*	*d*	95% CI
Get a better job	5.79	1.44	2.77	1.49	11.76 **	2.06	[2.51, 3.52]
Have good luck	5.80	1.40	3.48	1.52	9.03 **	1.59	[1.81, 2.82]
Receive CNY 1000 as a gift from a stranger	5.73	2.01	3.21	1.54	7.96 **	1.39	[1.89, 3.14]
Accidentally find a CNY 100 bill on the sidewalk	4.94	1.03	4.18	1.23	3.81 **	0.68	[0.37, 1.16]
Be very fortunate in life	5.29	1.18	3.28	1.33	9.07 **	1.60	[1.57, 2.45]
Always “be in the right place at the right time”	5.47	1.25	2.92	1.37	11.16 **	1.94	[2.10, 3.01]
Spend no night in a hospital for 10 years	4.54	1.72	3.90	1.54	2.24 *	0.39	[0.08, 1.20]
Win a lottery	4.74	1.22	3.88	1.55	3.46 **	0.62	[0.37, 1.35]
Earn a lot of money	4.86	1.40	3.33	1.41	6.22 **	1.09	[1.04, 2.02]
*Average: positive events*	5.24	0.66	3.44	0.95	12.44 **	2.20	[1.51, 2.09]
Fall victim to a brutal assault	3.57	1.54	6.57	1.77	−10.41 **	−1.82	[−3.57, −2.43]
Contract HIV through blood transfusion	3.29	1.78	4.52	1.64	−4.15 **	−0.72	[−1.83, −0.65]
Be victim of a natural disaster	4.54	1.64	4.92	1.44	−1.37	−0.24	[−0.91, 0.17]
Be bullied by colleagues	3.40	1.77	6.03	1.96	−8.05 **	−1.42	[−3.28, −1.99]
Find wallet was stolen	4.46	1.15	5.54	1.35	−4.96 **	−0.87	[−1.52, −0.65]
Die in a car accident	3.56	1.69	4.74	1.40	−4.37 **	−0.76	[−1.72, −0.65]
Have bad luck	4.39	1.28	5.70	1.42	−5.60 **	−0.98	[−1.79, −0.85]
Be cruelly murdered	3.14	1.69	4.90	1.97	−5.50 **	−0.96	[−2.39, −1.13]
Lose a loved one in a car accident	3.99	1.59	4.90	1.43	−3.46 **	−0.60	[−1.22, −0.39]
*Average: negative events*	3.81	1.04	5.31	0.82	−9.22 **	−1.60	[−1.82, −1.18]

*Note.* ** *p* < 0.01, * *p* < 0.05.

## Data Availability

The datasets used and analyzed during the current study are available from the corresponding author on reasonable request.
